# Three-Dimensional Atomic Force Microscopy for Sidewall Imaging Using Torsional Resonance Mode

**DOI:** 10.1155/2018/7606037

**Published:** 2018-07-19

**Authors:** Lu Liu, Jianguo Xu, Rui Zhang, Sen Wu, Xiaodong Hu, Xiaotang Hu

**Affiliations:** State Key Laboratory of Precision Measurement Technology & Instruments, Tianjin University, Tianjin 300072, China

## Abstract

This article presents an atomic force microscopy (AFM) technique for true three-dimensional (3D) characterization. The cantilever probe with flared tip was used in a home-made 3D-AFM system. The cantilever was driven by two shaking piezoceramics and oscillated around its vertical or torsional resonance frequency. The vertical resonance mode was used for upper surface imaging, and the torsional resonance mode was used for sidewall detecting. The 3D-AFM was applied to measure standard gratings with the height of 100 nm and 200 nm. The experiment results showed that the presented 3D-AFM technique was able to detect the small defect features on the steep sidewall and to reconstruct the 3D topography of the measured structure.

## 1. Introduction

The size of electron devices continues to decrease due to the development of semiconductor technology and the use of new materials. For example, the first 5 nm test chip was announced by IBM in 2017 [1], and the sub 10 nm era of CMOS technology has already started [2–4].

To improve the quality of the semiconductor devices, true three-dimensional (3D) metrology of microstructures is important. But there are still some difficulties in measuring critical dimension (CD) such as the width and sidewall, which was cited in International Technology Roadmap for Semiconductors (ITRS) as early as 2003 [5].

Scanning electron microscopy (SEM), confocal laser scanning microscopy (CLSM), atomic force microscopy (AFM), and optical scatterometry are the main topography measurement technologies in the semiconductor industry. Each method has its own advantages and limitations. Among these measurement techniques, AFM offers direct and almost nondestructive measurements of the shape of nanostructures with both high lateral and vertical resolution. However, the conventional AFM can only provide 2.5D topography information, because the cone-shaped probe is not able to contact the lateral surface of wall-like structure. For the true 3D characterization, several groups have developed three-dimensional atomic force microscopy (3D-AFM) or critical dimension atomic force microscopy (CD-AFM).

There are two possible ways to achieve 3D-AFM. One is known as the tilting method, in which the lateral surface is detected by tilting the sample or the probe. With the tilted components, the conical or pyramidal-shaped AFM tip can directly detect the steep sidewalls in the conventional scanning mode. For instance, a “tilted sample” AFM has been developed by Fouchier et al. [6], a 3D-AFM with rotatable Z scanner head has been designed by Pack Corporation [7], and several “tilted probe” AFMs have been developed by Xie et al., Jayanth et al., and Schuler et al. [8–11]. Xie et al. also reported a dual-probe AFM with two tilted optical fiber probes with relatively long tips [12, 13]. Each fiber probe had its own laser detecting systems and could work independently. The facing sides of a wall structure could be scanned by the two probes, respectively.

The full 3D profile of a wall-like structure can be reconstructed by stitching the images obtained at different tilting angles. However, this method can hardly be applied to detect high aspect ratio trench since the lateral side of the tip may contact the edge of the trench when the tilt angle increases.

Taking an alternative approach, cantilevers with special tip geometries (e.g., flared tip, sharpened tip, and carbon nanotube tip) are used to scan steep sidewalls. AFM cantilevers with special tips have been applied in different measurement ways. Murayama et al. have developed a multiangle step in method with a sharpened tip [14, 15]. In their method, the tip probed the surface point-by-point at different angles in a static mode. Dai et al. have developed a vector approach probing (VAP) method with a flared tip [16–18]. This method is similar to the step in method. At each measurement point, the probe was moved towards the surface until the tip-sample interaction force met a desired value, and then the probe was withdrawn from the surface. In Dai et al.'s design, the probe worked in both dynamic mode and static mode. A three-step scanning strategy was used in the AFM system, and it allowed individual configuration of scan settings according to different morphologies. Yasutake et al. developed sampling intelligent scan mode (SIS mode) with an aligned carbon nanotube (A-CNT) tip [19, 20] for sidewall measurement. In the SIS mode, the probe vibrated with small amplitude at its resonance frequency. The A-CNT tip mounted on a standard AFM silicon cantilever had high aspect ratio and robustness, which made it possible to measure the steep walls. But during the assembly of CNT tip, the angle and length of CNT were difficult to control. An improperly mounted CNT tip might cause instability of the scanning.

In this article, we present an amplitude modulation 3D-AFM system for the sidewall measurement. This system alternatively scans in vertical resonance mode (VR mode, referred as tapping mode) and torsional resonance mode (TR mode) with a flared tip. The free end of the tip has an extended geometry that enables probing of steep sidewalls. In the VR mode, the flared tip probes the surface vertically. This mode is used to obtain the top and bottom maps and find the position of the sidewalls. In the TR mode, the flared tip horizontally probes the sidewall and obtains the lateral profiles. In our design, the feedback loop maintains the amplitude constant while the tip is either scanned in VR mode or TR mode.

## 2. Method

### 2.1. Overall Structure

**Figure 1:**
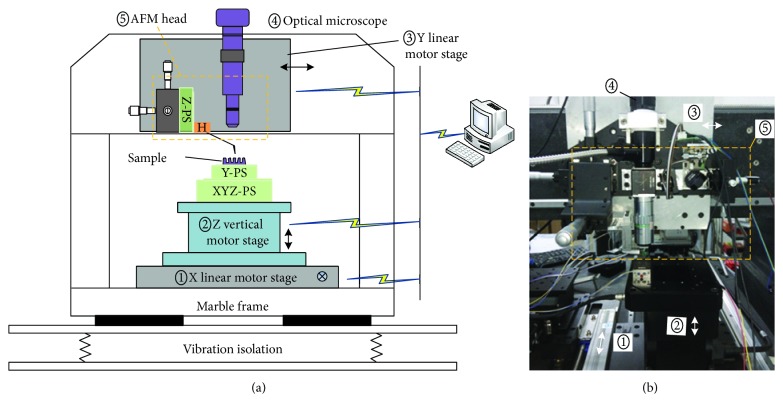
Schematic (a) and photo (b) of the 3D-AFM.

Figure 1 shows a schematic and photo of the 3D-AFM introduced in this article. The system is supported by a marble frame and laid on a vibration isolator. The system consists of an optical microscope, an AFM head, three motor stages, and a group of piezoelectric scanners. The optical microscope is used for locating the laser spot on the cantilever and positioning the sample. Three motor stages are used for coarse positioning of the tip relative to the sample. The piezoelectric scanners include two single-axis piezoelectric scanners (Z-PS and Y-PS) and one three-axis piezoelectric scanner (XYZ-PS). The Z-PS is included in the head part and drives the cantilever probe to scan in vertical direction. The Y-PS is mounted on the XYZ-PS, and it works in combination with the XYZ-PS as the sample stage. All the scanners are nanopositioning stages from PI (Physik Instrumente, Germany). The closed-loop travel ranges of the Y-PS, Z-PS, and XYZ-PS are 12 *μ*m, 12 *μ*m, and 100 × 100 × 20 *μ*m^3^.

For the upper surface measurement, the AFM system is operated in VR mode. In this mode, the XYZ-PS drives the sample to perform raster scanning in XY plane, and the Z-PS works as the feedback scanner to track the topography in a vertical direction. The Y-PS is in standby state. To measure the sidewall, the TR mode is applied. This mode was originally developed for frictional force measurement on relative soft surface [21, 22]. When the AFM is operated in TR mode, the XYZ-PS moves the sample in XZ plane to do the raster scanning, and the Y-PS works as the feedback scanner to track the features on the sidewall structure of the sample. The Z-PS changes to standby state.  Table 1 shows the activate scanners in different modes.

**Figure 2:**
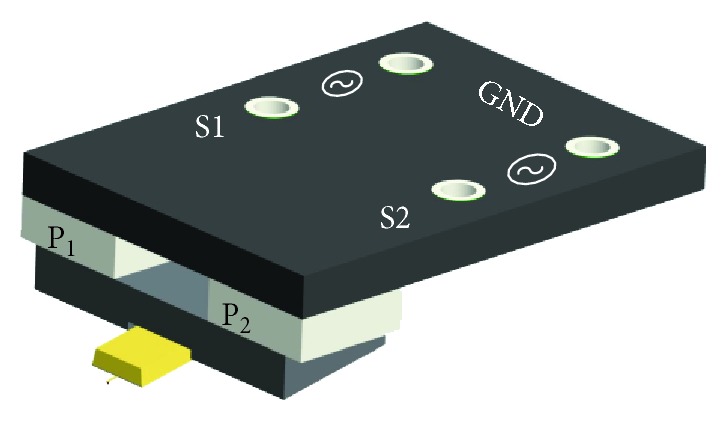
Diagrammatic drawing of the probe holder of the 3D-AFM.

The different oscillation modes of the cantilever are realized with two shaking piezo plates fixed in the probe holder as shown in Figure 2. These two piezo plates (P_1_ and P_2_) can be driven separately. When they are driven in phase at the first resonance frequency of the cantilever along the vertical direction, the cantilever probe works in VR mode. When the two shaking piezo plates are driven with inverse phase at the higher resonance frequency of the cantilever along the lateral direction, the TR mode can be activated. This method is similar to the technique introduced in Dai et al.'s work [16].

### 2.2. OBD System in the AFM Head

Like most AFMs, the optical beam detection (OBD) system, which is also called the optical lever system, is employed in the present 3D-AFM to detect the deformation and oscillation of the cantilever.

**Figure 3:**
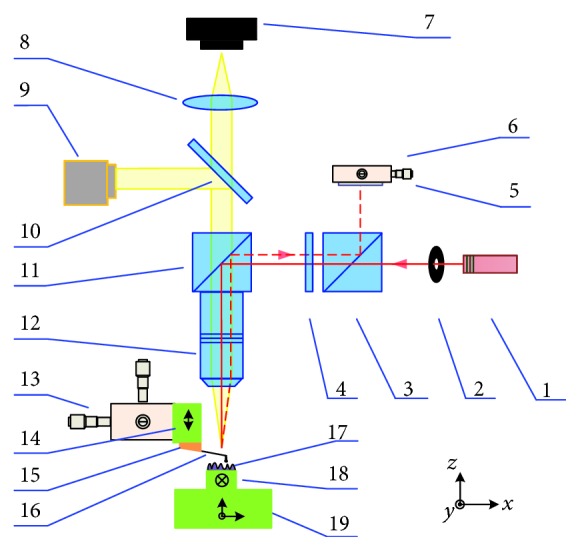
Schematic drawing of the designed OBD system with an optical microscope. One laser diode, 2 aperture, 3 polarization beam splitter, 4 quarter-wave plate, 5 quadrant photodetector (QPD), 6 dual-axis manual stage, 7 CCD camera, 8 tube lens, 9 LED illuminator, 10 beam splitter, 11 pellicle beam splitter, 12 objective lens, 13 three-axis manual stage, 14 single-axis piezoelectric scanner (Z-PS), 15 probe holder, 16 cantilever probe, 17 sample, 18 single-axis piezoelectric scanner (Y-PS), and 19 three-axis piezoelectric scanner (XYZ-PS).

Figure 3 shows the schematic of the optics in our AFM system. A laser diode module (CPS532, 532 nm, THORLABS, USA) with a mounted collimator lens is used as the laser source. The collimated laser beam goes through a polarization beam splitter and a quarter-wave plate and then be coupled into the optical microscope system via a pellicle beam splitter. The objective lens (M Plan Apo 20×, Mitutoyo, Japan) focused the laser on the backside of the cantilever near its free end. The spot size is less than 10 *μ*m. The reflected beam from the cantilever is also collected by the same objective lens and directed by the pellicle beam splitter, the quarter-wave plate, and the polarization beam splitter and finally reaches the quadrant photodiode detector (QPD). The deflection and torsion of the cantilever result in the motion of the laser spot along the vertical and lateral axes of the QPD (S5980, Hamamatsu Photonics, Shizuoka, Japan). The typical cut-off frequency of the QPD is about 25 MHz. The initial position of the laser spot on the QPD can be adjusted via a dual-axis manual stage. By using a three-axis manual stage, the position of the Z-PS relative to the objective lens can be adjusted. The alignment of the focused laser spot and the cantilever is also performed in this way.

### 2.3. Electronic Control System

**Figure 4:**
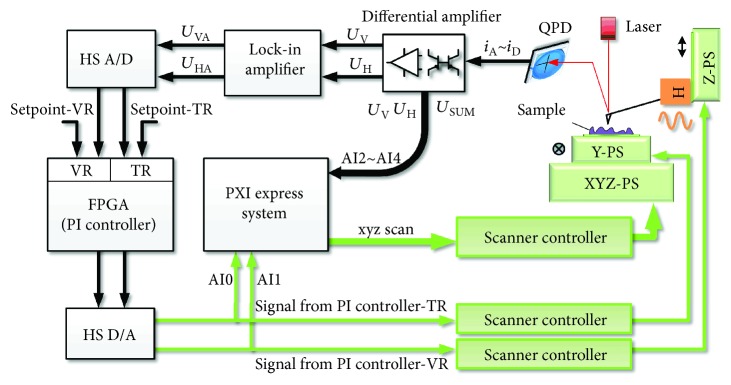
Schematic diagram of the electronic system of the 3D-AFM.

Figure 4 shows a schematic diagram of the electronic system of the 3D-AFM. The feedback control is based on a FPGA platform. A commercially PXI Express system (PXIe-1062Q, PXIe-6363, National Instruments) with 32 analog input (AI) and 4 analog output (AO) channels is employed for data acquisition, raster scanning generation, and image processing. A lock-in amplifier with integrated sine wave generator is applied to drive and detect the oscillation of the cantilever probe.

To reduce the delay of the analog signal processing, a current mode circuit is applied to obtain the sum signal (*U*_SUM_) and the normalized vertical and horizontal signals (*U*_*V*_, *U*_*H*_), which are proportional to the deflection and torsion of the cantilever [23]. Here, the photo currents of the four cells of QPD are denoted by *i*_A_, *i*_B_, *i*_C_, *i*_D_. Thus *U*_SUM_ = *R*_*s*_ · (*i*_A_ + *i*_B_ + *i*_C_ + *i*_D_), *U*_V_ = *R*_*V*_ · (*i*_A_ + *i*_B_ − *i*_C_ − *i*_D_)/*i*_SUM_, and *U*_H_ = *R*_*H*_ · (*i*_A_ + *i*_D_ − *i*_B_ − *i*_C_)/*i*_SUM_. All these signals are acquired by the PXI Express system through three AI channels and transferred to the PC via USB2.0 port for real-time observation. *U*_V_ and *U*_H_ are also sent to the lock-in amplifier for demodulation. The VR and TR oscillation amplitudes (*U*_VA_, *U*_HA_) of the cantilever are transferred to the FPGA for feedback control via two high-speed AD converters.

A simple proportion-integral-differential (PID) controller is used in the feedback loop. The digital output of the controller is converted to analog signal by a DA converter and transferred to the controller of Y-PS or Z-PS according to the operation mode.

## 3. Measurement Strategy and Results

### 3.1. Probe

**Figure 5:**
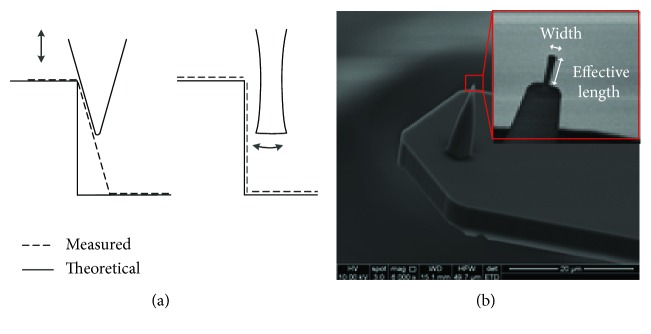
The influence of the different tips (a) and SEM image of the CDR-130C tip (b). The inset is a zoom in of the flared tip.

The tip effect is a common issue of AFM.  Figure 5(a) shows that the tip with different shapes leads to different results. The flared tip can reduce the artificial caused by the conventional cone-shaped tip when measuring steep sidewall [24].

**Figure 6:**
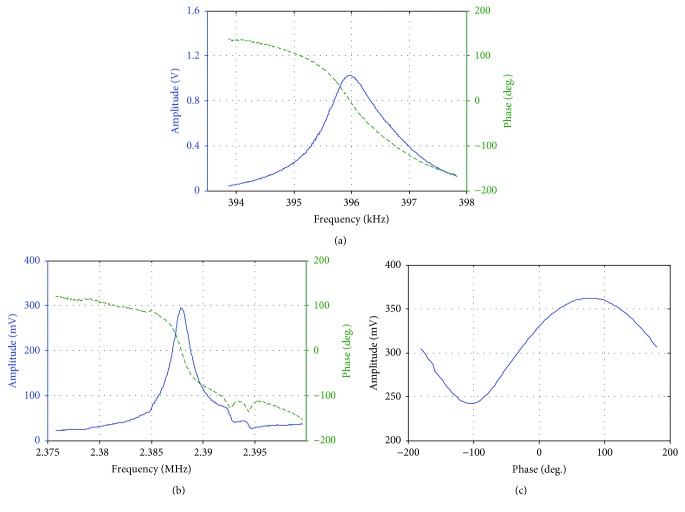
Frequency and phase sweeping results of CDR-130C. (a) Frequency sweeping for VR mode. (b) Frequency sweeping for TR mode. (c) Phase sweeping for TR mode.

A CDR-130C probe (Bruker, USA) with flared tip was used in the following experiments. This tip had an effective length of 700–900 nm, and the width was 110–140 nm. By frequency and phase sweeping, the vertical resonant frequency and torsional resonant frequency of this cantilever probe were determined as 395.8 kHz (Figure 6(a)) and 2.387 MHz (Figure 6(b)), respectively. Since the performance of the two shaking piezo plates was not exactly consistent, the actual phase difference between the two piezos in torsional resonance mode deviated from 180° as shown in the result of phase sweeping.

### 3.2. Detection Sensitivity Calibration

**Figure 7:**
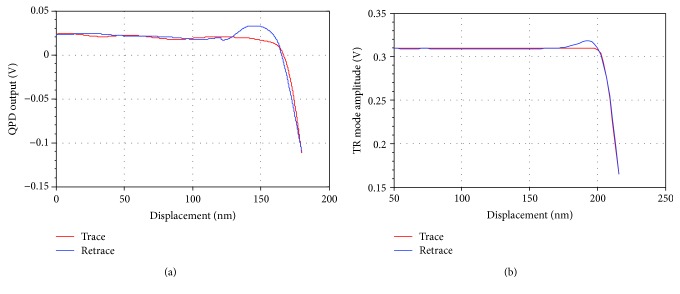
Voltage-displacement curve of horizontal probing. (a) The horizontal deformation-displacement curve based on the static mode (the slope of the retrace curve is 7 mv/nm). (b) The oscillation amplitude-displacement curve based on the TR mode (the slope of the retrace curve is about 12.7mv/nm).

The OBD sensitivity is based on the relationship between the QPD output and the tip or sample displacement. This parameter depends on many factors such as the position of the laser spot on the cantilever and the geometry of the cantilever. The vertical sensitivity calibration is a very common process which will not be discussed here. For the horizontal sensitivity calibration, a rigid sample with a trench structure was used. Before the calibration, a regular TR mode scan was performed. Then the tip was located into the trench and stopped beside the edge. The sample was moved horizontally by the Y-PS, and the tip was pushed from lateral by the sidewall of a trench. The horizontal signal of QPD and the corresponding displacement of Y-PS were simultaneously recorded as shown in Figure 7. The lateral sensitivity was determined by the slope of the voltage-displacement curve.

Figure 7(a) shows the horizontal deformation-displacement curve recorded in static mode, namely the cantilever did not vibrate during calibration.  Figure 7(b) shows the amplitude-displacement curve recorded in TR mode, namely the cantilever was in TR oscillation while being pushed by the sample. The results show that the TR mode was more stable and sensitive than the static mode in both approaching and retracting processes. It indicates that an oscillated probe is more practical in sidewall measurement compared to a static probe.

### 3.3. Sample Measurement

To investigate the capability and performance of the 3D-AFM, a standard grating (100 nm height, SHS-01, AppNano) was measured. This grating structure was defined in thermally grown silicon dioxide on a silicon substrate with an optional layer of Cr, which was deposited to harden the surface.

**Figure 8:**
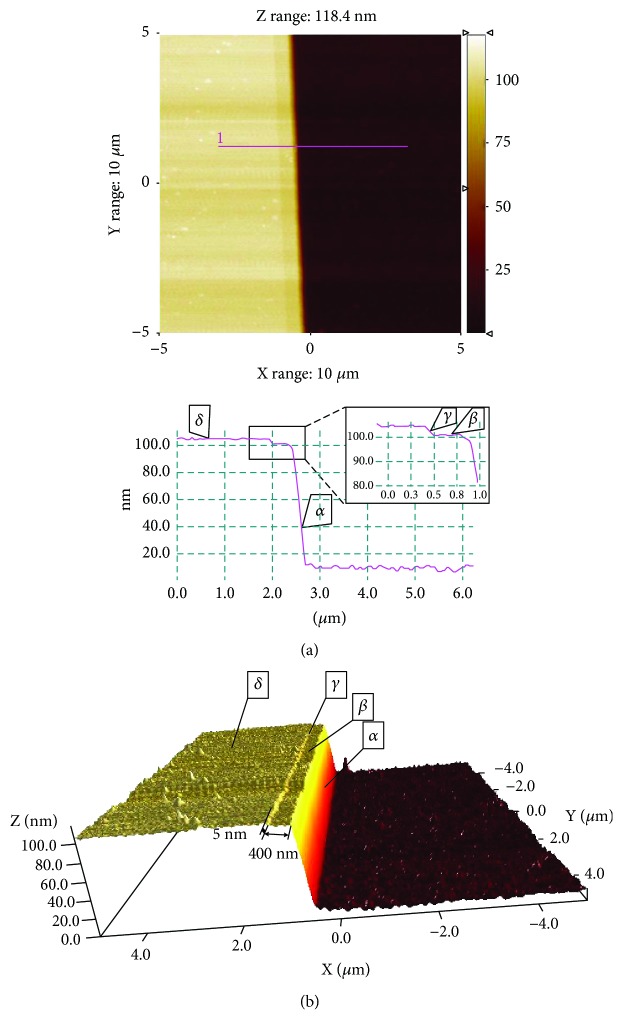
The topography of a standard grating was imaged with VR mode. (a) The AFM image and a cross-sectional profile at the marked position, and the inset in the black rectangle was the zoom of the small step. (b) 3D view by the VR mode of the step of 100 nm height.

For 3D image measurement, the 3D-AFM was firstly operated in VR mode at 395.8 kHz with the CDR-130C probe to obtain the top-bottom topography. The result is shown in Figure 8. The scan size and scan rate were 10 *μ*m × 10 *μ*m and 1 Hz, respectively. The pixels in the image were 512 × 512. According to the measurement, the grating height was 95.8 nm and the surface roughness 1.79 nm (Rq). The cross-section along the red line in Figure 8(a) is shown below. In the figure, a small step with the width of 400 nm and the depth of about 4 nm can be observed in the image.

**Figure 9:**
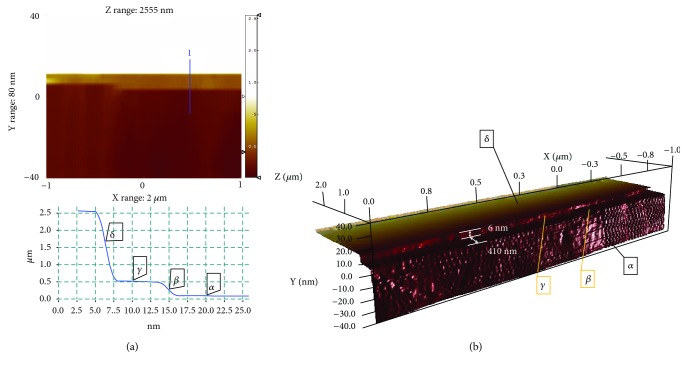
The sidewall topography of a standard grating was imaged with TR mode. (a) The AFM image and a cross-sectional profile at the marked position. (b) 3D view by the TR mode of the sidewall. The surfaces marked as *α*, *β*, *γ*, and *δ* were the same surfaces as in Figure 8.

In the second step, the 3D-AFM was operated in TR mode at 2.387 MHz with the same probe to obtain the sidewall topography, as shown in Figure 9. The scan size and scan rate were 2 *μ*m × 80 nm and 0.5 Hz, respectively. The pixels were 512 × 512. The roughness of the sidewall was 12.3 nm. The cross-section along the blue line in Figure 9(a) is shown below. The same step feature shown in Figure 8 can be observed again, which is clearly exhibited in the 3D view as shown in Figure 9(b). The size of this step feature was measured as 6 nm × 406 nm in this mode. In Figure 9(a), the upper half image is invisible. This is because the flared tip did not meet any structure above the upper surface of the grid and the Y-PS reached its limit position. So the recorded data were in saturation. Finally, with the top-bottom and sidewall topographies, a true 3D image was reconstructed. In the two figures, the same surfaces of the small step were marked as *α*, *β*, *γ*, and *δ*.

**Figure 10:**
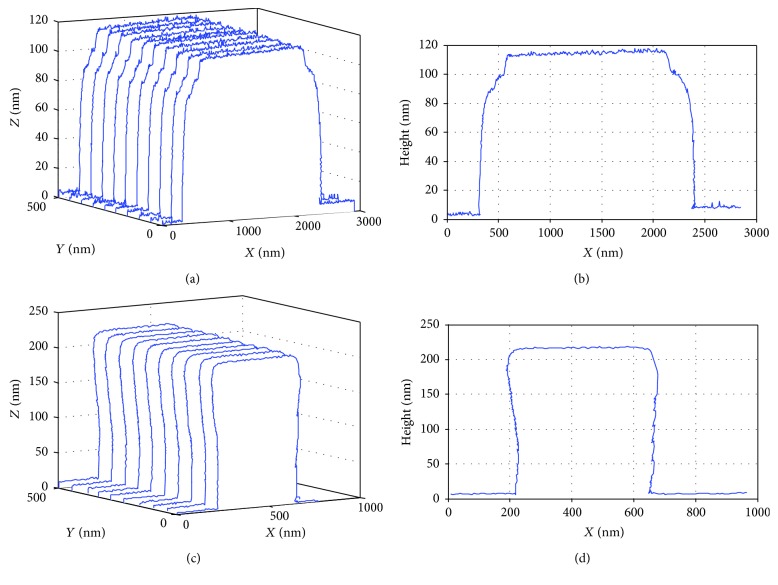
3D image of the different gratings by the 3D-AFM. 3D view of the STEP (a) and SNS (c) samples and their profile data (b, d).

Figure 10 shows two other measurement results. The samples were also standard grating (STEP-OX-0.1, AppNANO, USA; SNS-C12-1212, LightSmyth, USA).

## 4. Conclusions

We have developed a 3D-AFM for true three-dimensional measurements of nanostructures. In this 3D-AFM, the AFM probe is oscillated at vertical or torsional resonance frequency using two shaking piezos to configure the driving signals in different ways. In this way, the tip can tap the surface vertically with the VR mode and tap the sidewall laterally with the TR mode. To verify this method, a step height standard was measured with a flared tip by using VR and TR modes. In these modes, the same small steps were measured and the 3D-AFM shows reliable performance. However, with the increase of the sidewall depth, the torsional amplitude is not stable enough due to the increase interaction force between the sidewall and tip which has a long effective length.

## Figures and Tables

**Table 1:** Active scanners in different modes.

Axis	VR mode	TR mode
*x*	X scanner of XYZ-PS	X scanner of XYZ-PS
*y*	Y scanner of XYZ-PS	Y-PS
*z*	Z-PS	Z scanner of XYZ-PS

## Data Availability

The data used to support the findings of this study are included within the article.
